# A Real-Time Localization System for an Endoscopic Capsule Using Magnetic Sensors ^†^

**DOI:** 10.3390/s141120910

**Published:** 2014-11-05

**Authors:** Duc Minh Pham, Syed Mahfuzul Aziz

**Affiliations:** School of Engineering, University of South Australia, Mawson Lakes, South Australia 5095, Australia; E-Mail: mahfuz.aziz@unisa.edu.au

**Keywords:** *in vivo* tracking, magnetic localization system, real-time tracking, gastrointestinal tract, endoscopic capsule

## Abstract

Magnetic sensing technology offers an attractive alternative for *in vivo* tracking with much better performance than RF and ultrasound technologies. In this paper, an efficient *in vivo* magnetic tracking system is presented. The proposed system is intended to localize an endoscopic capsule which delivers biomarkers around specific locations of the gastrointestinal (GI) tract. For efficiently localizing a magnetic marker inside the capsule, a mathematical model has been developed for the magnetic field around a cylindrical magnet and used with a localization algorithm that provides minimum error and fast computation. The proposed tracking system has much reduced complexity compared to the ones reported in the literature to date. Laboratory tests and *in vivo* animal trials have demonstrated the suitability of the proposed system for tracking a magnetic marker with expected accuracy.

## 1. Introduction

Colon cancer, Crohn's disease, obscure GI bleeding, *etc.* are gastrointestinal diseases which are quite common in many countries around the world. Such gastrointestinal diseases are showing increased manifestations in Western societies as well [1]. The 2011 Australian Institute of Health and Welfare (AIHW) report indicated that colon cancer was the second largest cause of death during 2007 and 2010, with a burden of 15% and 19%, respectively [2]. These gastrointestinal diseases contribute to changing the functional characteristics of the gastrointestinal (GI) tract, commonly known as the gut. This phenomenon in turn results in the recurrence of these diseases with worse symptoms. On average, the human GI tract is approximately 9 m long. Traditional instruments such as fiber optic endoscopes and colonoscopes have an average length of about 2.5 m, and therefore cannot probe deeper into the GI tract [3]. Therefore, these instruments are unable to assist with investigations on the nature and causes of functional changes of the gut. Naturally, it will be of immense benefit if medical researchers can have access to technology that is able to reach the mid to distal regions of the GI tract for more precise exploration of the functions of these sections of the tract.

Wireless capsule endoscopes such as the SmartPill, PillCam, Norika *etc.* have made the examination of the GI tract much easier by eliminating the pain and discomfort associated with the insertion of conventional scopes [4]. However, these technologies lack the ability to examine the functional changes in the GI tract [5]. A possible solution is to deliver biomarkers at or near the affected spots in the gut and then monitor the response through clinical procedures such as breadth tests [6]. This will allow much of the biomarker to reach and act on the target area compared to oral delivery where bulk of the biomarker is absorbed in abdominal fluids. Targeted biomarker delivery is therefore expected to provide much better indication of the gut's functional changes through more credible breadth test response. Delivery of biomarkers to a specific region within the gut requires information about the real-time location of the capsule carrying the payload. This requires a system to track the capsule location in real-time. A variety of technologies can be considered for such *in vivo* tracking, for example, radio frequency (RF), magnetic and ultrasound technology. RF and ultrasound signals suffer large attenuations when passing through the human body [7,8], while magnetic signals are almost immune to *in vivo* attenuations [6]. For this reason magnetic technology is more suitable for *in vivo* capsule tracking.

Recent research literature has revealed that many *in vivo* magnetic tracking systems have been proposed to date [9–15]. These tracking systems have a magnet placed inside the capsule. As the magnet travels down the gut, a number of magnetic sensors placed outside the body continually measure the magnetic field strengths. These magnetic field readings are used to determine the location of the capsule. The majority of the proposed systems use a large number of magnetic sensors, often placed in a two dimensional array. Although the accuracies obtained by some of these systems are in the range of millimeters the actual systems are impractical to use in a real application due to their complexity, namely, size, large number of sensors, bulk of cables, *etc*.

In this paper, we present a tracking system for an endoscopic capsule, which uses a small magnet placed inside the capsule for tracking. A new mathematical model and a tracking algorithm have been developed to determine the location of the capsule from the magnetic field strength readings. The proposed tracking system is much less complex than the tracking systems reported to date. Operation and accuracy of the proposed system have been verified using animal trials involving pigs. The low complexity and good tracking accuracy of the proposed system make it suitable for *in vivo* tracking of a magnetically marked endoscopic capsule. A concise paper with some preliminary results of the work has been introduced in [16]. In contrast, in this paper, we present the details of the mathematical model, tracking algorithm, details of animal trials with extensive results and comparisons.

## 2. Mathematical Model for Magnetic Field

The magnetic field produced by a magnet at a certain point is dependent on the distance of the magnet from that point and its orientation relative to that point. We can measure the magnetic field intensity and direction by placing a few magnetic sensors around the point in a suitable arrangement, and then calculate the magnet's distance and orientation parameters using an algorithm. In this section, a mathematical model for the magnetic field created by a permanent magnet is introduced and will be used to find the location and orientation parameters using an optimization algorithm. The optimization algorithm is selected based on the best possible calculation accuracy and lowest execution time.

### 2.1. Position and Orientation of the Magnet

**Figure 1.**
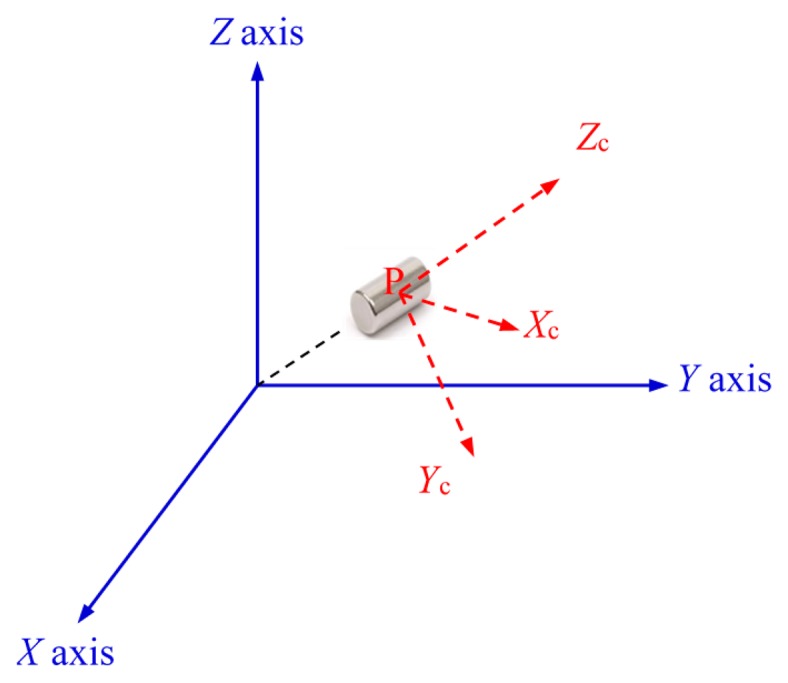
Position and orientation of the magnet in cartesian coordinate system.

The endoscopic capsule will contain a small cylindrical magnet, which has a finite length. This means that the magnet can be placed with its center at point P as shown in Figure 1 but its length orientated in different directions in space. To represent the magnet's orientation, we can attach a coordinate system to the magnet itself and then relate this coordinate system to the reference system. Figure 1 shows a coordinate system {*XcYcZc*} that has been attached to the magnet relative to the reference coordinate system {*XYZ*}. {*XcYcZc*} is a vector that specifies only the orientation of the magnet. The relationship between the two coordinate systems can be represented by a rotation matrix, which is the amount of rotation required to align the two systems.

### 2.2. Existing Magnetic Field Models

Review of relevant literature has revealed that a few mathematical models have been developed to describe the behavior of the magnetic field strength with distance. An empirical model developed in [6] is given as:

(1) $$D=\frac{55}{1-a.e^{-bx}}$$

where, *D* is the distance between the magnet and the sensor, *x* is the value of magnetic field intensity, while *a* and *b* are variables depending on the properties of the magnet used [6]. It addresses the issues with determining the magnetic field intensity when the magnet is at 90° and 180° orientations with respect to the sensor, however it does not incorporate the effects of all other orientation angles [6].

Another mathematical model used widely in existing magnetic tracking systems [10–12,17–19], is expressed as:

(2) $$B=K_{T}\left(\frac{3\left(H.\vec{R}\right)\vec{R}}{R^{5}}-\frac{H}{R^{3}}\right)$$

in which the experiment is conducted, *H* represents the orientation of the magnetic field and *R* is the distance between the magnet and the sensor.

### 2.3. Building a Mathematical Model for a Magnetic Dipole

**Figure 2.**
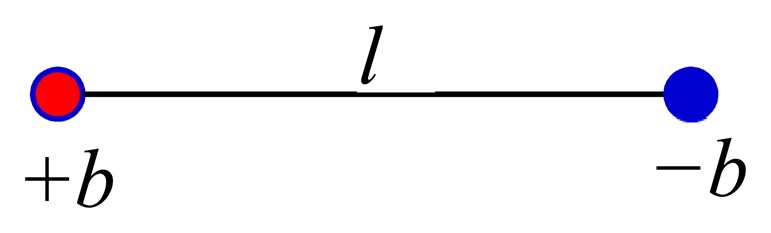
Two-charge dipole.

A dipole model, shown in Figure 2, consisting of two charges, +*b* and −*b*, separated by a distance *l*, produces the same field as a current loop *bl* = *IA* = |*μ*|, where *μ* is the magnetic dipole moment and is a vector [20]. Since the two models produce the same field, we can use the two-charge dipole model to determine the field.

**Figure 3.**
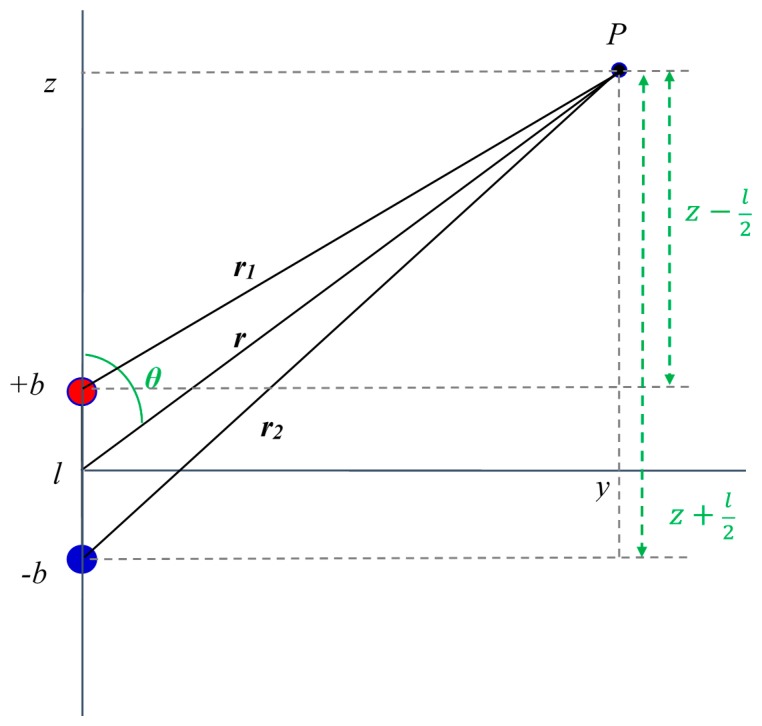
Distances of point P from the origin and the two charges.

We'll further assume that the dipole is aligned with the *z* axis. We want to find the field at point *P* as shown in Figure 3. P is s assumed to lie in the *y*-*z* plane. The field at point *P* will be the sum of the fields due to charges +*b* and −*b*. The distances of *P* from the origin and the two charges are:

(3) $$\begin{array}{c} r=\sqrt{z^{2}+y^{2}}\\ r_{1}=\sqrt{{\left(z-\frac{l}{2}\right)}^{2}+y^{2}}\\ r_{2}=\sqrt{{\left(z+\frac{l}{2}\right)}^{2}+y^{2}} \end{array}$$

For a point charge, if the radius vector at a particular location has angle *θ* with the *z* axis and the total field strength at that location is |*B*|, we have:

(4) $$B_{z}=\left|B\right|\cos\theta$$

Summing the fields of the two point charges, we have:

(5) $$\begin{array}{c} B_{z}=B_{z\left(+b\right)}+B_{z\left(-b\right)}\\ B_{z}=b\left[\frac{z-\frac{l}{2}}{r_{1}^{3}}-\frac{z+\frac{l}{2}}{r_{2}^{3}}\right] \end{array}$$

The size of the magnet is usually small, *l* ≪ *r*.

(6) $$\begin{array}{c} \frac{1}{r_{1}^{3}}=\frac{1}{\sqrt{{\left[(y^{2}+{\left(z-\frac{l}{2}\right)}^{2}\right]}^{3}}}\\ \frac{1}{r_{1}^{3}}=\frac{1}{\sqrt{{\left[(y^{2}+z^{2}-zl\right]}^{3}}}=\frac{1}{\sqrt{{\left[r^{2}(1-\frac{zl}{r^{2}})\right]}^{3}}}=\frac{1}{r^{3}}(1-\frac{3zl}{2r^{2}}) \end{array}$$

Putting (6) into (5), multiplying out, and discarding all terms containing *l*^2^, we obtain:

(7) $$B_{z}=\frac{bl}{r^{3}}\left[3\frac{z^{2}}{r^{2}}-1\right]=\frac{bl}{r^{3}}\left[3\cos\theta -1\right]$$

Similarly, to calculate the component *B_y_*, we start with the field for a point charge:

(8) $$B_{y}=\left|B\right|\sin\theta$$

Summing the fields of the two point charges, we get:

(9) $$\begin{array}{c} B_{y}=B_{y(+b)}+B_{y(-b)}\\ B_{y}=by(\frac{1}{r_{1}^{3}}-\frac{1}{r_{2}^{3}}) \end{array}$$

Putting (6) into (9) we get:

(10) $$B_{y}=\frac{by}{r^{3}}\left[\frac{3zl}{2r^{2}}+\frac{3zl}{2r^{2}}\right]$$

(11) $$B_{y}=\frac{3bl}{r^{3}}\frac{yz}{r^{2}}$$

(12) $$B_{y}=\frac{3bl}{r^{3}}\sin\theta \cos\theta$$

Finally, we put (7) and (12) together to obtain the total field at point *P*. If we use φ for the azimuth, the angle of rotation about the *z* axis, then, using the convention that the unit basis vectors are written with hats, we have:

(13) $$\vec{B}=\frac{3bl}{r^{3}}\left[\sin\theta \cos\theta \left(\cos\varphi \hat{\text{x}}+\sin\varphi \hat{\text{y}}\right)+(\cos^{2}\theta -\frac{1}{3})\hat{\text{z}}\right]$$

To express the field in polar coordinates, we write the Cartesian basis vectors in terms of the usual orthonormal polar basis:

(14) $$\begin{array}{c} \hat{x}=\sin\theta \cos\varphi \hat{r}+\cos\theta \cos\varphi \hat{\theta }-\sin\varphi \hat{\varphi }\\ \hat{y}=\sin\theta \sin\varphi \hat{r}+\cos\theta \sin\varphi \hat{\theta }-\cos\varphi \hat{\varphi }\\ \hat{z}=\cos\theta \hat{r}-\sin\theta \hat{\theta } \end{array}$$

In (13), coefficient on *x̂* is:

$$\begin{array}{c} \sin\theta \cos\theta \cos\varphi \left(\sin\theta \cos\varphi \hat{r}+\cos\theta \cos\varphi \hat{\theta }-\sin\varphi \hat{\varphi }\right)\\ =\cos\theta \left(\sin^{2}\theta \cos^{2}\varphi \hat{r}+\sin\theta \cos\theta \cos^{2}\varphi \hat{\theta }-\sin\theta \sin\varphi \cos\varphi \hat{\varphi }\right) \end{array}$$

In (13), coefficient on *ŷ* is:

$$\begin{array}{c} \sin\theta \cos\theta \sin\varphi \left(\sin\theta \sin\varphi \hat{r}+\cos\theta \sin\varphi \hat{\theta }-\cos\varphi \hat{\varphi }\right)\\ =\cos\theta \left(\sin^{2}\theta \sin^{2}\varphi \hat{r}+\sin\theta \cos\theta \sin^{2}\varphi \hat{\theta }-\sin\theta \sin\varphi \cos\varphi \hat{\varphi }\right) \end{array}$$

In (13), coefficient on *ẑ* is:

$$\begin{array}{c} (\cos^{2}\theta \frac{1}{3})\left(\cos\theta \hat{r}-\sin\theta \hat{\theta }\right)\\ =\cos\theta \left(\cos^{2}\theta \hat{r}-\sin\theta \cos\theta \hat{\theta }\right)-\cos\theta (\frac{1}{3}\hat{r}-\frac{1}{3}\tan\theta \hat{\theta }) \end{array}$$

Putting the above coefficients back in (13) and obtain the coefficient for each *r̂*, *θ̂*, φ̂, we have:

Coefficient on *r̂* is:

$$\begin{array}{c} \cos\theta \sin^{2}\theta \cos^{2}\varphi +\cos\theta \sin^{2}\theta \sin^{2}\varphi +\cos\theta (\cos^{2}\theta -\frac{1}{3})\\ =\cos\theta (\sin^{2}\theta \cos^{2}\varphi +\sin^{2}\theta \sin^{2}\varphi +\cos^{2}\theta -\frac{1}{3})\\ =\frac{2}{3}\cos\theta \end{array}$$

Coefficient on *θ̂* is:

$$\cos\theta \left(\sin\theta \cos\theta -\sin\theta \cos\theta +\frac{1}{3}\tan\theta \right)=\frac{1}{3}\sin\theta$$

Coefficient on φ̂ is:

cosθ(−sinθsinφcosφ+sinθsinφcosφ)=0

Finally, we put these coefficients into (13) to obtain the polar coordinate expression for the field for a dipole aligned on the *Z* axis:

(15) $$B=\frac{bl}{3r^{3}}\left[2\cos\theta \hat{r}+\sin\theta \hat{\theta }\right]$$

**Figure 4.**
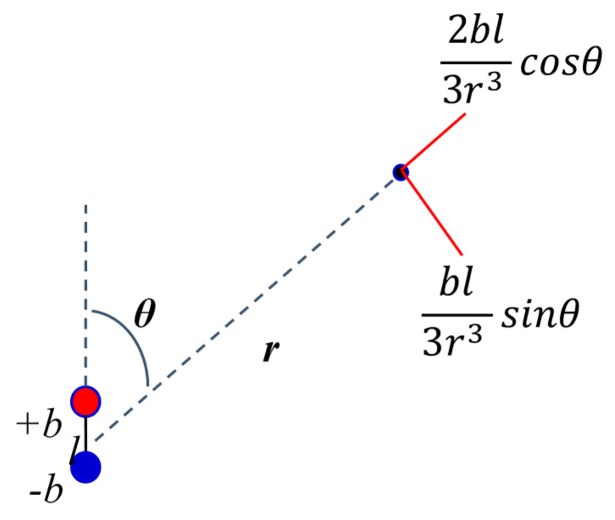
Field calculation angle and radial distance.

We can use Equation (15) to calculate the field with only two parameters, angle and radial distance. Using these assumptions, the field components are calculated as shown in Figure 4.

## 3. The Proposed Tracking System

In the proposed tracking system, a small permanent magnet (neodymium) is used as the source of a static magnetic field. The tracking system also consists of magnetic sensors with on-board microprocessors for data acquisition, data pre-processing and communication. The magnetic sensors are attached to a flat panel, which is to be placed in front of the gut to measure the magnetic field strength. The signals produced by each sensor are sent to its dedicated (on-board) microprocessor, which collects and stores the data and forward to a PC. A tracking algorithm running on the PC uses the magnetic signals from all the sensors and displays the calculated location of the magnet (capsule) in real-time.

### 3.1. Magnet

**Figure 5.**
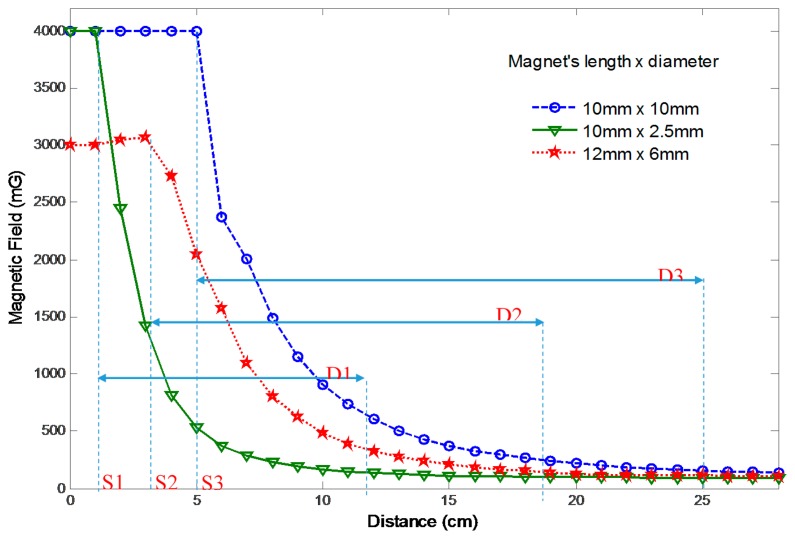
Characteristic curves for cylindrical magnets.

**Figure 6.**
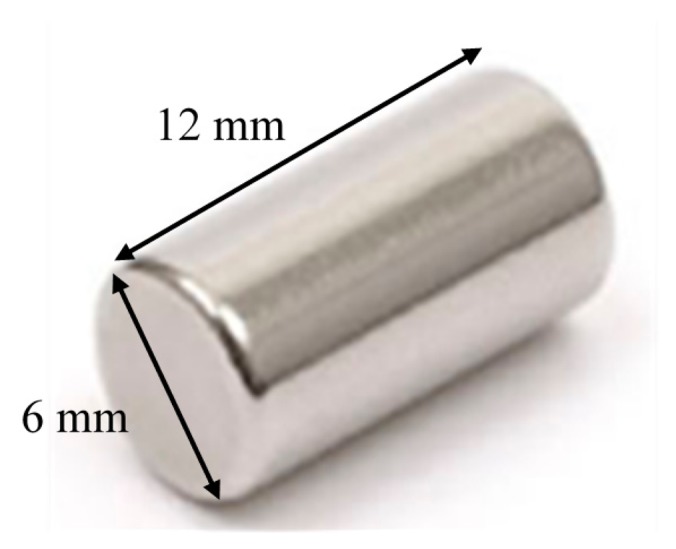
A 12 × 6 mm neodymium magnet.

For a magnetic tracking system, the selection of material, shape and size of the magnet is very important. It is understood that the rare earth neodymium magnets (Nd_2_Fe_14_B) possess the highest field to size ratio [6]. It is also known that the cylindrical or rod shaped magnets produce a higher magnetization in air compared to other shapes like disk, ring, sphere, tube, *etc.* [21]. In order to select the proper size of the magnet, experiments have been conducted to determine the characteristic curves of several cylindrical neodymium magnets of different sizes. Figure 5 shows the graphs of the magnetic field strength versus distance for magnets having sizes (Length × Diameter in mm) 10 × 2.5, 12 × 6 and 10 × 10. It is pertinent to mention that the magnetoresistive sensor provides the peak reading when the magnet is moved very close to the sensor. The magnetic sensor is in saturation in such a situation. The graphs indicate the points of saturation *S1*, *S2*, *S3* and the detection ranges *D1*, *D2*, *D3* for the three magnets respectively. From this, it is obvious that the 10 × 10 magnet has the highest detection range of almost 20 cm, however, its saturation point (*S3*) starts from a distance of 5 cm from the sensor. The saturation point *S1* for the 10 × 2.5 magnet is quite reasonable as it starts from about 1.5 cm from the sensor, but its detection range of 11 cm is insufficient. The detection range for the 12 × 6 magnet is almost 15 cm with the point of saturation (*S2*) starting from 4 cm. The anatomy of human gut suggests that if the average diameter of the body around the gut is 30 cm then the separation between the outer wall and the small intestine will always be greater than 4 cm from any side. So, it can be concluded from the above experiments that the magnet with size 12 × 6, as shown in Figure 6, is suitable for use with our tracking system.

### 3.2. Magnetic Sensors

The PNI RM3000 magnetic sensor suite has been used to measure the magnetic field strength. The RM3000 is an integrated magnetic field sensing module with highest accuracy in their class, a large magnetic field measurement range, high resolution, low power consumption, and large signal noise immunity under all conditions. It is also highly stable over temperature. Each RM3000 sensors suite has the ability to detect the magnetic field in three perpendicular axis (*X*, *Y*, *Z*) using three physical sensors. We identify them as SX, SY and SZ. The sensitivity of the sensors is inversely related to the sampling rate, *i.e.*, time required to take one measurement.

- At the highest sampling rate of 2.4 KHz/Axis, the sensor's resolution is ∼1.4 mG.
- At the lowest sampling rate of 300 Hz/Axis, the sensor's resolution is ∼0.15 mG.

In the proposed system, the sensor is set at the highest sensitive mode to achieve the highest Signal to Noise Ratio (SNR). The RM3000 sensor suite has a SPI interface for data communication. The SPI interface includes *CE* (chip enable), *MOSI* (master output, slave input), *MISO* (master input, slave output), *SCLK* (clock), *DRDY* (data ready) and *CLEAR*. To read the data from the RM3000 sensor, the microprocessor must read each axis separately. The SPI interface shifts out a 24-bit field measurement value for each of the three axis which represent the three orthogonal components of magnetic field density *i.e.*, *B_x_*, *B_y_* and *B_z_*. The resultant magnetic field *B*, its azimuth angle *θ* and elevation angle φ are calculated from these values using Equations (16)–Equation (18), respectively [22]:

(16) $$\left|B\right|=\sqrt{B_{x}^{2}+B_{y}^{2}+B_{z}^{2}}$$

(17) $$\theta =\tan^{-1}\left(\frac{B_{y}}{B_{x}}\right)$$

(18) $$\varphi =90-\cos^{-1}\left(\frac{B_{z}}{B}\right)$$

### 3.3. Processor Board and Communication Module

**Figure 7.**
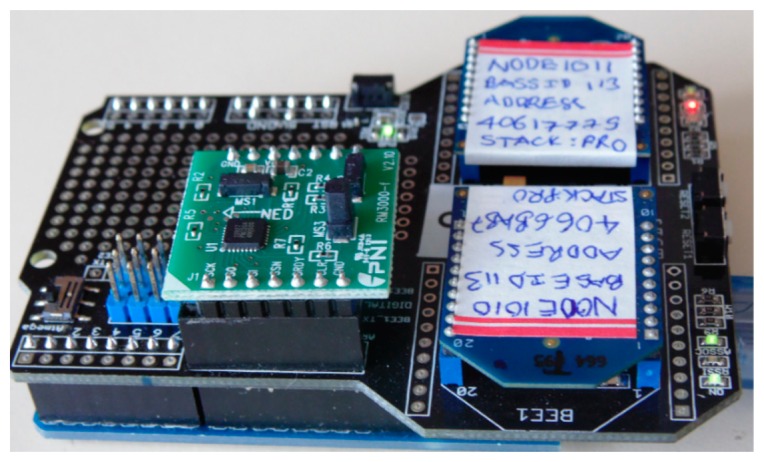
Microprocessor board with RM3000 sensor suite.

The PNI RM3000 magnetic sensors suite is connected to a microcontroller board via a daughter board. The microcontroller board is based on the ATmega328 microcontroller. It communicates with the RM3000 sensors via SPI interface. The received magnetic field intensity data can be stored in the internal memory of the microcontroller and transmitted to a PC for further processing. Communication between the microcontroller and the PC is via USB interface. Figure 7 shows the microcontroller board with RM3000 sensor suite.

### 3.4. Sensor Location

In order to correctly locate the magnet, we must know the field strength as well as the location where the sensor measured the field. This means that the exact location of each of the sensors must be known and this location must be fixed for all measurements. For this reason, the sensors are mounted on a flat plastic base so the sensors are on the same *x*-*y* plane. This base ensures that the locations of the sensors do not change and that their relative positions to each other are fixed. The base also provides information about the location of each sensor in 3D.

**Figure 8.**
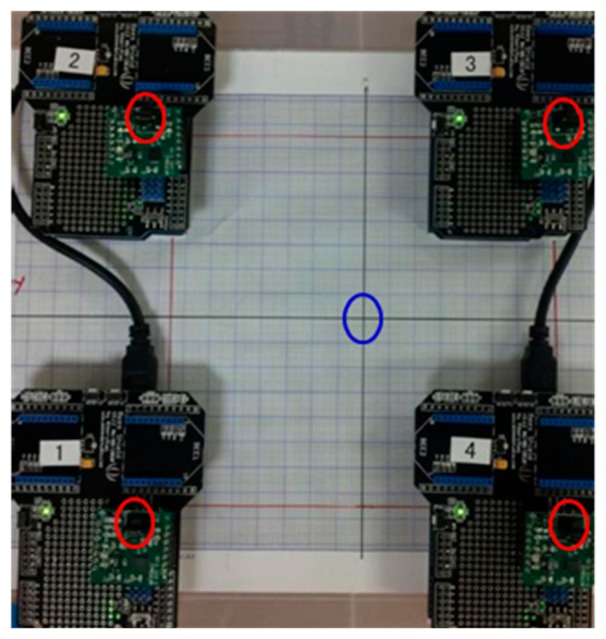
System setup with four 3-axis magnetic sensors.

The reference point (0,0,0) of the system is marked with a blue circle as shown in Figure 8. Location of each sensor is marked with a red circle. From this arrangement, the locations of all four sensors were identified as shown in Table 1.

As stated previously, each sensor board has three physical sensors: SX, SY and SZ. The sensor SX measures the magnetic field along the *X*-axis, SY measures the field along the *Y*-axis, and SZ measures the field along the *Z*-axis. Locations of SX, SY and SZ are shown in Table 1.

### 3.5. Communication of Magnetic Data to PC

The microcontroller is programmed to control the timing of all the signals. It also processes the data and transmits to a PC via a USB hub. The present system is set up using wired communication between the sensors and the PC. In future, wireless communication can be used using a reliable protocol similar to the one presented in [23].

### 3.6. Sensor Calibration and Noise Level

Before putting the system into use, calibrations are required for determining the sensitivity of the sensors, adjusting the sensor's reading and normalizing the measurements between the sensor boards. It is important to ensure that each of the three-axis sensors on each sensor board is calibrated properly. The criterion used for calibration of the individual axis sensors (*X*,*Y*,*Z*) is the fact that the overall magnitude of the field |B| (as per Equation (16)) should not vary irrespective of the orientation of the sensor board. The Earth's magnetic field is used as a reference field.

To calibrate the individual axis (*X*,*Y*,*Z*), we aligned the sensor board to a fixed orientation and took the field readings from each individual axis. Then, we rotated the sensor board to swap the axis (for example, swap *X* axis with *Y* axis) and took another measurement from each individual axis. From the readings of all possible axis swapping, we were able to determine the scaling factor needed to calibrate the *Y*-axis and *Z*-axis to the reference *X*-axis. The next step of calibration was to normalize the measurements across the four sensor boards. We chose one sensor as the reference sensor and scaled the readings of the other three sensors to the field measured by the reference sensor.

**Figure 9.**
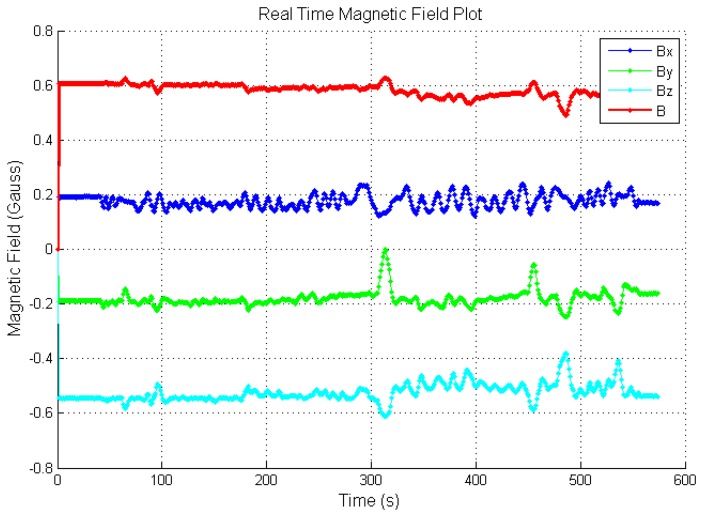
Uncalibrated sensor data.

**Figure 10.**
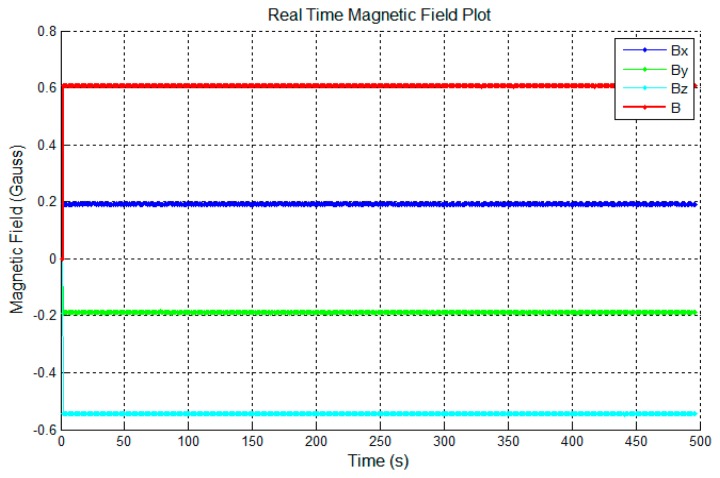
Sensor data after calibration.

Figure 9 shows the Earth's magnetic field measured before calibration and Figure 10 shows the same field after calibration. *B_x_*, *B_y_*, *B_z_* are the magnetic field along *X*, *Y* and *Z* axis. *B* is the overall magnetic field. Clearly, the field magnitude (Figure 10) is much more stable after calibration.

**Figure 11.**
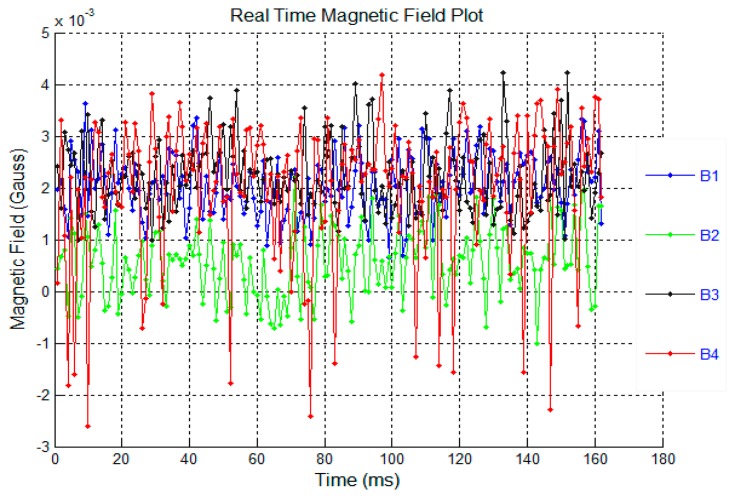
Noise level of the sensor system.

**Figure 12.**
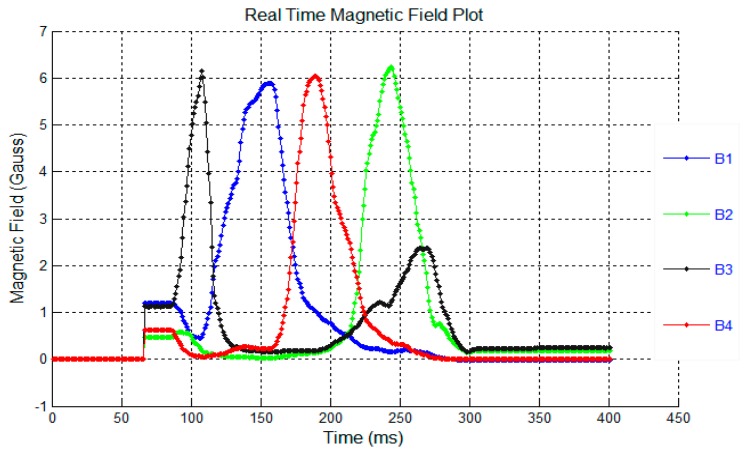
Signal level of the sensors system in Gauss.

The signal to noise ratio of the system was also determined. Figure 11 shows the white background noise and Figure 12 shows the signal level read from a 6 × 12 mm magnet at a distance of 30 mm. The signals B1–B4 in Figures 11 and 12 show the overall magnetic field of sensor 1–4 in the sensor system. Obviously, the noise range of +/− 3 G is very small compared with the signal level of 6 G.

## 4. Tracking Algorithm

### 4.1. Magnet Localization

The magnetic marker contained in the swallowable endoscopic capsule can be tracked inside the GI tract using multiple sensors placed outside the body around the gut region at pre-determined locations. Four 3-axis magnetic sensors are used and their positions on the plastic base were shown in Table 1. The problem can be considered as one of finding the location and orientation of a magnet whose calculated magnetic field most closely matches the field measured by the sensors at their respective locations. The calculation of the magnetic fields are done using mathematical models presented in Section 2. Finding the location and the orientation which make the difference between the measured field and the calculated field minimum is a non-linear optimization problem for the six parameters which describe the position and orientation of the magnet (see Section 2.1).

Several nonlinear minimization algorithms have been reported in literature, e.g., Powell's [24], Downhill Simplex [25], DIRECT [26], and Levenberg-Marquardt Algorithm [27]. The algorithms normally require an initial guess of the parameters, or their bounds, to begin the search for the minimum. If the initial guess is too far away from the solution then the algorithms may fail to provide a solution because there may be many local minima. Besides that, the minimization algorithm should be fast enough to enable real-time implementation and should also be robust to noises in the sensor data. In [17], the authors have thoroughly evaluated the performance of the above algorithms with respect to the localization error, orientation error and computation time required. From their findings, the Levenberg-Marquardt Algorithm (LMA) has the minimum localization and orientation errors. This is particularly so when the error levels of the initial guess of locations are confined within a 20 × 20 × 20 cm^3^ space, which is the same as the space we have used for testing our tracking system in the laboratory. Moreover, the paper [17] also reports that the Levenberg-Marquardt Algorithm requires the minimum computation time for converging to a solution. Therefore we have decided to use the LMA for implementing the proposed tracking system.

### 4.2. Expected Tracking Accuracy

The proposed tracking system is intended to provide information on the location of an endoscopic capsule in the GI tract to assist with targeted delivery of biomarkers inside the tract. In traditional oral delivery, much of the biomarker gets absorbed in abdominal fluid before it reaches the intended GI region. If the target area is in the mid to distal regions of the GI tract, then very little of the biomarker delivered orally reaches near the intended spot. Hence gastroenterologists consider oral delivery methods to be problematic for effective functional characterization or diagnosis of problems in the mid to distal regions of the GI tract. Targeted delivery near the region of interest is expected to make such studies much more effective. From this perspective, compared to orally administered methods, biomarker delivery within 5–10 cm of the GI region of interest will be more than sufficient to extract more pronounced response to the biomarker. A tracking system that provides the location information with this level of accuracy (within 10 cm error) is deemed to be adequate for the above purpose.

### 4.3. Localization Results

**Figure 13.**
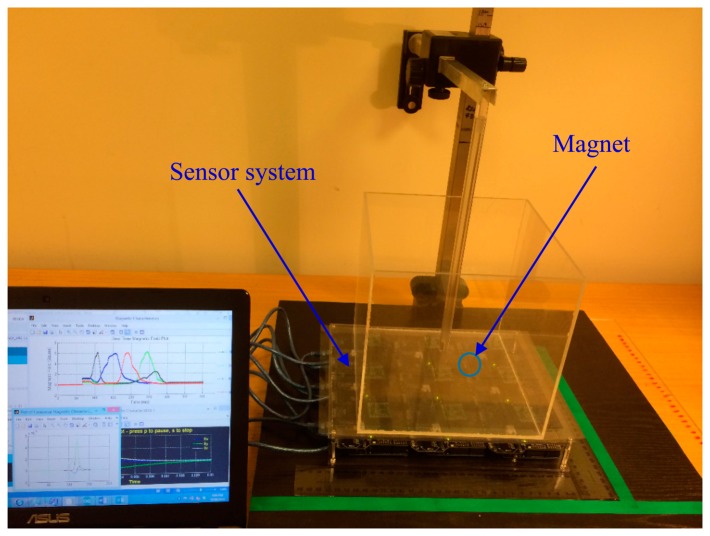
*In vitro* experimental setup.

The tracking accuracy is evaluated by conducting *in vitro* tests in the laboratory. The magnet is placed at various locations and orientations within a measurement volume of 20 × 20 × 20 cm^3^ above the sensor plane. These locations include the worst case (boundary) conditions around the edges of the measurement volume. Figure 13 shows the *in vitro* experimental set up. Staring from an initial guess, the tracking algorithm keeps on searching for the location of the magnet until it finds a location for which the magnetic field values calculated using the proposed mathematical model closely match the actual magnetic field values measured by the sensors. This means that for this location the difference (error) between the magnetic field values calculated by the algorithm and the actual magnetic field values is the minimum. When the algorithm converges in this manner, the corresponding location coordinates reported by the algorithm are compared with the actual coordinates, *i.e.*, the known physical locations of the magnet. The difference is the localization error or tracking accuracy. The following observations were made from the *in vitro* tests:

- For each location, tests were conducted multiple times to determine the error of the system which was found to be approximately 5 mm. In other words, the localization accuracy of the proposed system is approximately 5 mm.
- Four sensors are enough to obtain the required accuracy (less than 10 cm error) for our application. If more sensors are used, the accuracy is likely to increase, however the complexity and computation time will increase too. The tracking accuracy is higher when the magnet is located in the central area above the sensor plane.

**Figure 14.**
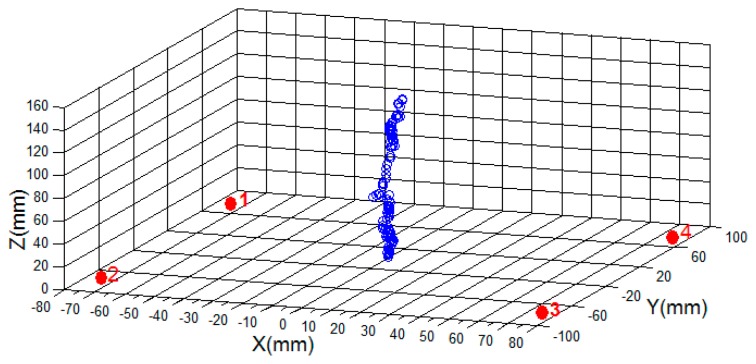
Up-down trajectory test.

Figure 14 shows some position tracking performed in the laboratory using the four sensors and the 6 × 12 mm magnet. This example shows a test with the magnet moving up and down above the sensor plane. The magnet's trajectory is shown in red and the sensor locations are shown in blue.

### 4.4. Field Test with Animal Model

**Figure 15.**
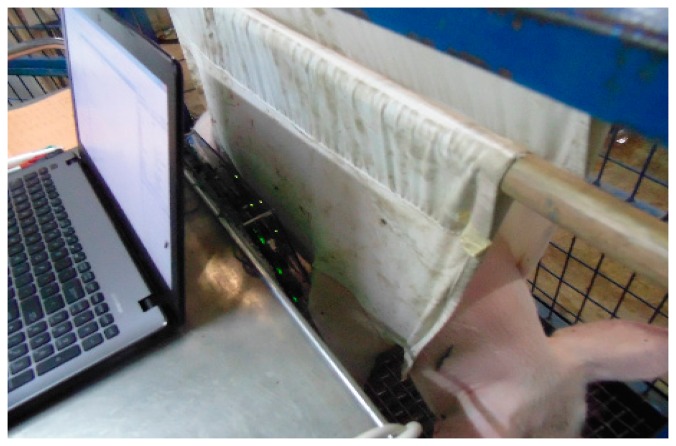
Pig under test with the tracking system.

**Figure 16.**
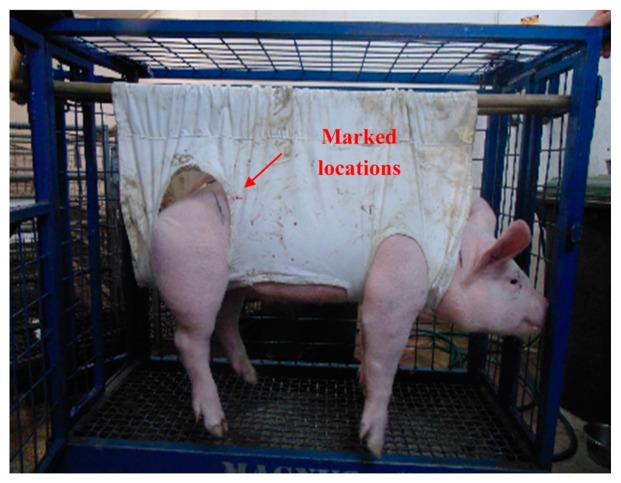
Location of the magnet is determined and marked on the pig.

**Figure 17.**
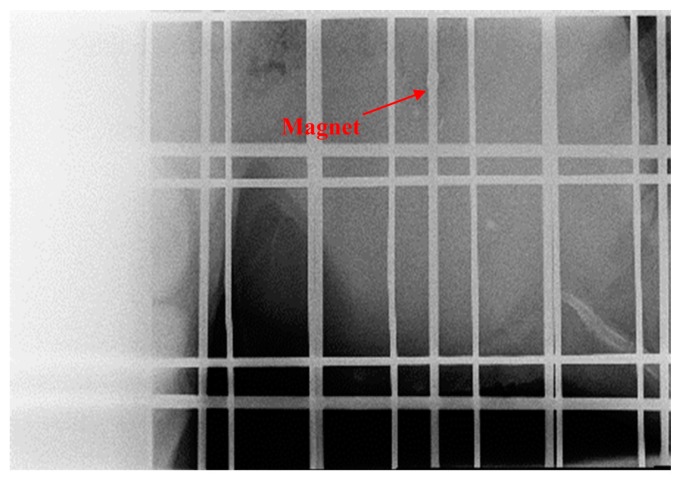
Location of the magnet is verified by X-Ray.

The proposed tracking system has been used to track a magnet inside a capsule which was swallowed by pigs. Location of the magnet inside the pig's body is determined by the tracking system. Figure 15 shows the pig under test with the tracking system placed on its side. Four X-rays were taken during the tracking to cross check the magnet's location obtained from the tracking system with those shown on the X-rays. Figure 16 shows the location of the magnet marked on the body of the pig in 2D plane as obtained from the tracking system. This location is verified by X-ray as shown in Figure 17. X-ray is taken intermittently and therefore it does not provide real-time tracking. However, the X-ray provides visual indication of the location of the magnet in 2D for validation against the 2D location from the tracking system.

In order to determine the real-time performance of the proposed tracking system, we have recorded the computation time taken for each of the LMA searches during the animal trial. The computation time is measured from the start of each search to the time when one satisfied location is found. At the end of the animal trial, we have found that the system's average computation time for one search is 7.518 ms which is equivalent to 133 calculations per second.

## 5. Performance Comparison

*In vivo* tracking systems can be compared on the basis of accuracy, cost, complexity, portability and patient's comfort *etc*. Table 2 shows a comparison of the proposed system with existing *in vivo* magnetic tracking systems reported to date. The 5 mm error reported in Table 2 for the proposed tracking system is the localization error obtained from laboratory measurements conducted for various magnet locations in 3D within a measurement volume of 20 × 20 × 20 cm^3^. The method of measurement and calculation of error was described in Section 4.2.

Section 4.2 outlined that the localization accuracy expected of a tracking system for targeted delivery of biomarkers in the GI tract is 5–10 cm. The proposed tracking system exceeds this expectation by a very large margin, the tracking accuracy being 5 mm when tested in a volume of 20 × 20 × 20 cm^3^ in the laboratory. A few millimeters of deviation will not make a noticeable impact on the expected response when compared to orally administered methods. Despite this, a comprehensive comparison of complexity and accuracy of the proposed system is provided in Table 2 for the sake of completeness.

For some existing systems, information on the measurement volume is missing or given partially [13,14,17,18,28]. In [6], the magnet used has a volume that is 1.4 times that of the one used in the proposed system and also testing is conducted in a measurement volume that is eight times smaller. Yet the average error in [6] is two times that of the proposed system. In [11], the error is slightly lower, however it uses 25 sensors as opposed to only four used in the proposed system. In [12], the error is 20% lower than that of the proposed system, however the measurement volume is eight times smaller and uses four times the number of sensors as compared to the proposed system. In [13], although the error is much smaller, the information on measurement volume is incomplete. The systems in [14,18,19,22,28] use a significantly large number of sensors to inhibit their practical use. The error reported in [15,17] are comparable to that of the proposed system but they both use four times the number of sensors compared to the proposed system. Also, [15] uses a complex arrangement of two permanent magnets placed 10 cm apart, and therefore is impractical to use in a swallowable endoscopic capsule. In [29], although the error is lower than that of the proposed system it uses four times the number of sensors and the measurement volume is 8 times smaller than that of the proposed system.

From the above it is clear that the proposed system provides a significant reduction in complexity (number of sensors) for *in vivo* tracking. This has been possible due to the development of an accurate mathematical model of the magnetic field distribution around a permanent cylindrical magnet and an improved real-time tracking algorithm. Its tracking accuracy is more than adequate for the intended purpose of delivering biomarkers in the proximity of a target location within the GI tract. The tracking accuracy compares well, and in many cases better, than those of majority of the tracking systems reported to date.

## 6. Conclusions

A new *in vivo* magnetic tracking system has been presented in this paper to determine the real-time location of an endoscopic capsule. Using only four 3-axis magnetic sensors, the proposed tracking system achieves high localization accuracy. Although there are quite a few tracking systems reported in literature, the majority of them are far too complex for practical implementation. Unlike other tracking systems, the proposed system has been used in animal trials involving pigs, and its operation has been satisfactorily verified. The low complexity and high tracking accuracy of the proposed system make it suitable for *in vivo* tracking of a magnetically marked endoscopic capsule.

## Figures and Tables

**Table 1.** Sensor locations.

**Sensor #**	**SX (x,y,z) mm**	**SY (x,y,z) mm**	**SZ (x,y,z) mm**
**1**	(−75,−75,0)	(−75,−75,0)	(−75,−75,0)
**2**	(75,−75,0)	(75,−75,0)	(75,−75,0)
**3**	(75,75,0)	(75,75,0)	(75,75,0)
**4**	(−75,75,0)	(−75,75,0)	(−75,75,0)

**Table 2.** Comparison with other magnetic tracking systems reported to date.

**Article**	**Sensors**	**Vol (cm^3^)**	**Algorithm**	**Error (mm)**
Proposed	4	20 × 20 × 20	LMA	5
[6]	3	10 × 10 × 10	?	10
[11]	25	30 × 30 × 30	Linear	4.2
[12]	16	12 × 12 × 10	LMA	4.0
[13]	16	24 × 24 × ?	LMA	2.0
[14]	80	? × ? × ?	LMA	2.1
[15]	16	50 × 50 × 50	LMA	4.5
[22]	64	40 × 25 × 40	LMA	3.9
[17]	16	24 × 24 × ?	LMA	4.6
[18]	49	? × ? × ?	LMA	3.3
[19]	64	50 × 50 × 50	LMA	5.0
[28]	64	? × ? × ?	LMA	3.0
[29]	16	10 × 10 × 10	LMA	3.3

? indicates unknown dimension or missing information; LMA stands for Levenberg-Marquardt Algorithm.
